# Hereditary chronic pancreatitis

**DOI:** 10.1186/1750-1172-2-1

**Published:** 2007-01-04

**Authors:** Jonas Rosendahl, Hans Bödeker, Joachim Mössner, Niels Teich

**Affiliations:** 1 Medizinische Klinik und Poliklinik II, Universität Leipzig, Germany

## Abstract

Hereditary chronic pancreatitis (HCP) is a very rare form of early onset chronic pancreatitis. With the exception of the young age at diagnosis and a slower progression, the clinical course, morphological features and laboratory findings of HCP do not differ from those of patients with alcoholic chronic pancreatitis. As well, diagnostic criteria and treatment of HCP resemble that of chronic pancreatitis of other causes. The clinical presentation is highly variable and includes chronic abdominal pain, impairment of endocrine and exocrine pancreatic function, nausea and vomiting, maldigestion, diabetes, pseudocysts, bile duct and duodenal obstruction, and rarely pancreatic cancer. Fortunately, most patients have a mild disease. Mutations in the PRSS1 gene, encoding cationic trypsinogen, play a causative role in chronic pancreatitis. It has been shown that the PRSS1 mutations increase autocatalytic conversion of trypsinogen to active trypsin, and thus probably cause premature, intrapancreatic trypsinogen activation disturbing the intrapancreatic balance of proteases and their inhibitors. Other genes, such as the anionic trypsinogen (PRSS2), the serine protease inhibitor, Kazal type 1 (SPINK1) and the cystic fibrosis transmembrane conductance regulator (CFTR) have been found to be associated with chronic pancreatitis (idiopathic and hereditary) as well. Genetic testing should only be performed in carefully selected patients by direct DNA sequencing and antenatal diagnosis should not be encouraged. Treatment focuses on enzyme and nutritional supplementation, pain management, pancreatic diabetes, and local organ complications, such as pseudocysts, bile duct or duodenal obstruction. The disease course and prognosis of patients with HCP is unpredictable. Pancreatic cancer risk is elevated. Therefore, HCP patients should strongly avoid environmental risk factors for pancreatic cancer.

## Disease name/synonyms

Hereditary chronic pancreatitis

## Definition/diagnostic criteria

### Genetic definition

Already in 1952 Comfort and Steinberg were first to recognize that chronic pancreatitis may accumulate in selected families suggesting a genetic background [1]. Thereafter, hereditary chronic pancreatitis (HCP) was defined as an autosomal dominant disease with a penetrance of approximately 80%. However, in the daily clinical setting the inheritance pattern cannot be determined in some cases. In 1996 several groups mapped a gene for HCP to chromosome 7 [2-4]. In the same year, Whitcomb and colleagues identified an R122H mutation in the cationic trypsinogen gene (*PRSS1*) [5]. Several other mutations were described subsequently (A16V, D22G, K23R, N29I, N29T, R122C) [6-12]. Until now, the R122H and N29I mutations of the *PRSS1 *gene have been identified as the most common disease associated mutations [5-7].

**Figure 1:**
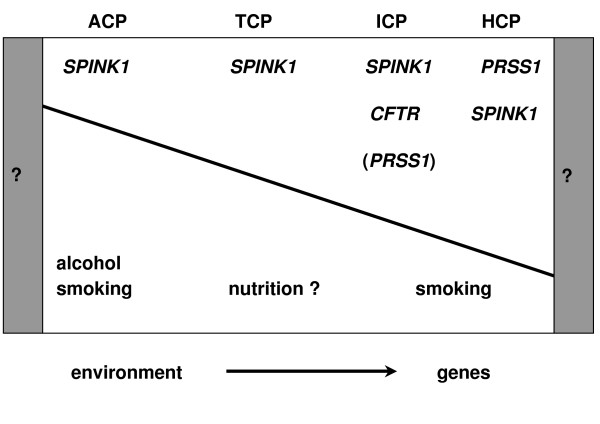
Diagrammatic illustration of genetic and environmental factors with their suspected influence on the pathogenesis of chronic pancreatitis. Abbreviations: ACP = alcoholic chronic pancreatitis, TCP = tropical calcific chronic pancreatitis, ICP = idiopathic chronic pancreatitis, HCP = hereditary chronic pancreatitis; abbreviations of the genes see within the text (According to Witt, [85]).

In the last decade, several authors identified associations of chronic pancreatitis (idiopathic and hereditary) to other genes, such as the anionic trypsinogen (*PRSS2*), the Serine Protease Inhibitor, Kazal type 1 (*SPINK1*) and the cystic fibrosis transmembrane conductance regulator (*CFTR*) [13-16]. On the other hand, environmental factors as smoking, alcohol consumption or the lack of antioxidants were assumed to be important manifestation factors, even in HCP [17-20] (Figure 1).

The definition of HCP as a classic autosomal dominant disorder represents the current knowledge. However, the criteria of the diagnosis of HCP have been changing over the years and are currently different in the various clinical centres. In the recently published Europac study, the diagnosis of hereditary pancreatitis was made on the basis of two first-degree relatives or three or more second-degree relatives, in two or more generations with recurrent acute pancreatitis, and/or chronic pancreatitis for which there were no precipitating factors. Cases in which these strict criteria were not met, but more than one affected family member was identified, mostly within the same generation, were classified as familial chronic pancreatitis [21]. However, the diagnostic value of this classification is questionable. Therefore, we define HCP if the patient has no other detectable cause of chronic pancreatitis and if he/she has one first or second degree relative with proven chronic pancreatitis. An international consensus is needed in the near future to classify affected families unambiguously.

## Clinical definition and diagnostic criteria

### Clinical definition

Chronic pancreatitis in adults is defined as a relapsing or continuing inflammatory disease of the pancreas characterized by irreversible morphological changes, upper abdominal pain and, in some patients, permanent impairment of exocrine function, endocrine function, or both [22]. The clinical course during an acute attack may range from mild edematous to severe necrotizing inflammation of the pancreas. The resulting morphological changes can be summarized as irregular sclerosis with focal, segmental, or diffuse destruction of the parenchyma. Frequently dilatations, strictures, or intraductal plugs can be seen in the pancreatic duct system. Initially, chronic pancreatitis is characterized by a recurrent stage of acute pancreatitis (early stage CP) passing over to progressive pancreatic dysfunction and/or pancreatic calcification (late stage CP).

Noteworthy in children the cardinal symptom is recurring, suddenly appearing epigastric pain. Contrary to adults, enduring pain is not a common clinical finding in children. Other symptoms are nausea, vomiting and abdominal pressure pain. Children partially develop pancreatic insufficiency with steatorrhoea and insulin-dependent diabetes, but these complications normally occur later than in patients with chronic alcoholic pancreatitis.

### Diagnostic criteria

Diagnosis of chronic pancreatitis is made by clinical findings, a typical medical and family history, imaging methods and pancreatic function tests. The use of invasive function tests (secretin test, pancreozymin-secretin test) is declining over the last years. In recent reviews it was stated that invasive function tests in combination with pancreatic calcification are still the gold standard in the diagnosis of chronic pancreatitis, but these tests are laborious and costly [23-26]. Non-invasive tests (faecal chymotrypsin, PABA-, pancreolauryl- and faecal elastase test) are insufficient for the detection of minor or moderate insufficiency as a result of their restricted sensitivity compared to the pancreozymin-secretin test. As a consequence, function tests are of restricted value, particularly in early stage chronic pancreatitis [26,27]. A pragmatic and reasonable treatment is the supplementation of pancreatic enzymes *ex juvantibus *in patients with chronic pancreatitis and suspected exocrine insufficiency.

So far no imaging test has been established for the diagnosis of early stage chronic pancreatitis. In patients with late stage chronic pancreatitis several imaging methods (ERCP, MR, MRCP, ES, CT, US, abdominal x-ray) are sufficient to detect typical morphological changes (e.g. duct alterations, calcification).

### Epidemiology

There are no data regarding the incidence or prevalence of chronic hereditary pancreatitis or of chronic pancreatitis in children. The incidence of chronic pancreatitis of any cause is expected to be about 3.5–10 per 100.000 inhabitants and year in Europe and the USA [28,29].

## Clinical description

### Clinical characteristics

Alcoholic chronic pancreatitis and HCP exhibit essentially identical clinical laboratory results and histopathological or morphological features. Remarkably, HCP manifests typically at an earlier age and pancreatic calcification and diabetes mellitus are less frequent complications in comparison to chronic alcoholic pancreatitis. Certainly estimable is the fact that most investigated subjects with the N29I or R122H *PRSS1 *mutation had mild disease or were asymptomatic [17,30].

Our investigations revealed no difference in the age of onset between carriers of the these mutations. The median age of onset was 11 years in the N29I and 10 years in the R122H group, respectively. Only 4% of our patients had severe chronic pancreatitis with exocrine and endocrine insufficiency, pancreatic calcification and duct dilatation as well as hospitalizations due to pancreatitis. In general, half of the mutation carriers had little or no complaints or complications [17]. A European study revealed a mean age of onset of 10 years and 14.5 years for affected carriers of the *PRSS1 *mutations R122H and N29I, respectively, but showed no mutation-dependent differences in complications such as exocrine or endocrine insufficiency or increased pancreatic cancer risk [21]. Data on the frequencies of acute pancreatitis attacks and pain in patients with HCP are not available to date. Chronic pancreatitis presents with a wide range of pain from mild to severe and from intermittent to persistent. Interestingly, endocrine insufficiency can regress over time, which is in contrast to current believe, that pancreatic diabetes is an irreversible sign of pancreatic failure [31]. However, prospective investigations of the course of diabetes in patients with HCP are lacking so far.

### Hereditary chronic pancreatitis and pancreatic cancer

As shown in an investigation of 8 patients with pancreatic cancer in a cohort of 246 HCP patients, the lifetime risk of pancreatic cancer is about 50-fold higher than in the control population and corresponds with 1 per 1066 person-years. It is only 20-fold elevated in patients with chronic alcoholic pancreatitis [32,33]. In our cohort of 101 HCP patients (25 N29I carrier, 76 R122H carrier), pancreatic cancer was diagnosed in 3 patients with the R122H mutation with a median of 23 years after the onset of pancreatitis. This corresponds to a rate of about 1 per 1200 person-years among affected R122H carriers [17]. Obviously, the data basis for the estimation of the pancreatic cancer risk in patients with *PRSS1 *associated HCP is small. The largest clinical investigation in the pre-genetic era, however, revealed no pancreatic cancer in 72 patients from 7 families [19]. Taken together, the pancreatic cancer risk in HCP patients with a *PRSS1 *mutation seems to be elevated – with an uncertain relative risk increase. Today, there is no generally accepted protocol for screening HCP patients for early pancreatic cancer. However, affected mutation carriers should be strongly advised to stop smoking, as it is an additional risk factor for pancreatic cancer [34].

## Other genes associated to chronic pancreatitis

### *SPINK1 *mutations in chronic pancreatitis

Witt and colleagues described at first an association between mutations of the serine protease inhibitor, Kazal type 1 (*SPINK1*) and chronic pancreatitis [14]. SPINK1 is a potent protease inhibitor thought to be a specific inactivation factor of intrapancreatic trypsin activity. During incubation of equimolar quantities of trypsin and SPINK1 the formation of a covalent bond between the catalytic serine residue of trypsin and the lysine carboxyl group of the reactive site of SPINK1 is carried out. After prolonged incubation, trypsin activity reappears over time. This is explainable by the fact that SPINK1 is degraded by trypsin [35]. The most frequently found *SPINK1 *mutation is N34S. This mutation was predominantly found in patients with idiopathic chronic pancreatitis. Further investigations showed an association of *SPINK1 *mutations and alcoholic chronic pancreatitis as well as tropical chronic pancreatitis [36-41]. Since 1–2% of controls carry the N34S mutation, this mutation alone seems to be insufficient to explain the pathogenesis of chronic pancreatits in mutation carriers. Moreover, a functional analysis of recombinant SPINK1 with the N34S mutation showed an unchanged function of N34S SPINK1 as well as an unchanged trypsin susceptibility [42]. This indicates that mechanisms other than the conformational change of N34S might underlie the predisposition to chronic pancreatitis in mutation carriers.

### *CFTR *mutations in chronic pancreatitis

Cystic fibrosis (*OMIM 219700*) is an autosomal recessive disorder with an incidence in whites of approximately 1 in 2500 live births. In 1989, *CFTR *(*OMIM 602421*) was identified as the underlying gene. In 1998, Sharer and colleagues and Cohn and colleagues were able to show an association of *CFTR *mutations with chronic pancreatitis [15,16]. This association is pathophysiologically comprehensible since 1–2% of patients with cystic fibrosis suffer from chronic pancreatitis [43,44]. The variety of pancreatic disorders in cystic fibrosis range from complete loss of exocrine and endocrine function to almost normal pancreatic function. So far more than 1500 mutations of the *CFTR *gene have been described [45]. According to their effect the mutations are split up in five or six classes (I-V/VI) [46,47]. In cystic fibrosis, the most common mutation is F508del, accounting for approximately 66% of all mutated alleles [48]. Interestingly, the clinical course of cystic fibrosis can be variable in patients carrying the same mutations, indicating the influence of environmental and maybe other genetic factors.

Recently published studies confirmed the association of chronic pancreatitis and *CFTR *mutations, but until now the underlying mechanisms leading to the development of chronic pancreatitis are poorly understood [49-54]. One of the main findings in all investigations is the detection of mostly rare *CFTR *mutations showing a different spectrum of detected mutations than in cystic fibrosis and congenital bilateral aplasia of the vas deferens (CBAVD). Some authors state that compound heterozygous *CFTR *carriers have a distinct elevated risk for the development of chronic pancreatitis, which is even higher when an additional *SPINK1 *mutation is present [51,54].

However, the role of some *CFTR *mutations has to be reconsidered since Rohlfs et al. demonstrated that the mutation I148T in Exon 4, which was classified as a severe cystic fibrosis causing mutation, is not associated to cystic fibrosis. According to their data the complex allele 3199del6 and I148T seems to be the relevant factor [55]. In summary, *CFTR *mutations alone are not sufficient for the pathogenesis of chronic pancreatitis in most patients and further studies are needed to elucidate the role of CFTR in the pathogenesis of chronic pancreatitis.

### *PRSS2 *mutations in chronic pancreatitis

In an actual study of 2466 patients with chronic pancreatitis (including 1857 with hereditary or idiopathic pancreatitis) and 6459 controls by Witt et al., the G191R variant of the anionic trypsinogen was over represented in controls (32 vs. 220, odds ratio 0.37; *P *= 1.1 × 10^-8^). The analysis of the recombinantly expressed G191R variant revealed a complete loss of trypsin activity due to the introduction of a novel tryptic cleavage site that renders the enzyme hypersensitive to autocatalytic proteolysis. Taken together, the G191R variant of PRSS2 mitigates intrapancreatic trypsin activity and thereby plays a protective role against chronic pancreatitis [13]. This is the first study demonstrating a mutation with a protective effect in chronic pancreatitis.

### Aetiology and biochemical analysis of disease associated *PRSS1 *mutations

Classic HCP seems to follow an autosomal dominant inheritance with incomplete penetrance and highly variable disease expression. As stated above the results of research done within the last decade implicate a more complex inheritance pattern.

**Figure 2:**
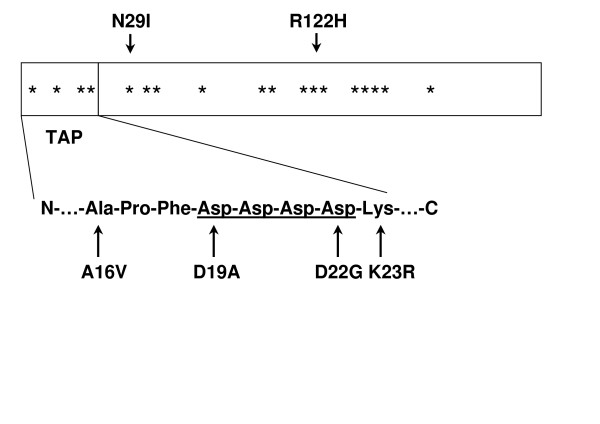
Linear map of pancreatitis associated mutations within the primary structure of the human cationic trypsinogen. The amino-acid positions affected by the pancreatitis-associated *PRSS1 *mutations are denoted by asterisks (*). The positions of the most frequent N29I and R122H mutations are indicated. The blue call-out demonstrates the sequence of the trypsinogen activation peptide (TAP) and the mutations found in this region. The highly conserved tetra-aspartate motif in the activation peptide is underlined and bold.

The *PRSS1 *mutations are located in three clusters within the trypsinogen sequence: in the TAP (trypsinogen activation peptide), in the N-terminal part of trypsin or in the longest peptide segment not stabilized by disulfide bonds between Cys64 and Cys139 (Figure 2).

**Figure 3:**
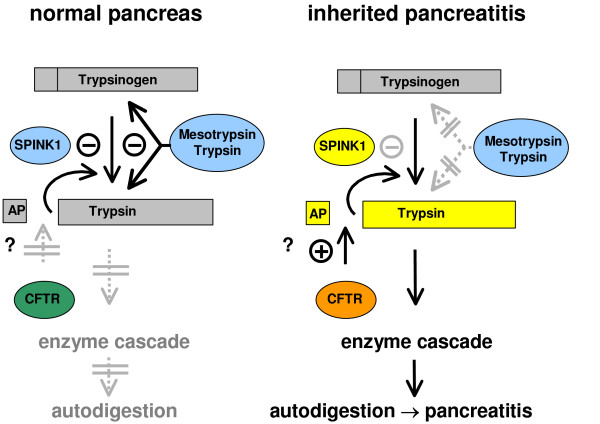
Model of inherited pancreatitis. In the normal pancreas (left) trypsin that is prematurely activated within the pancreas is inhibited by SPINK1 and in the second line by trypsin and mesotrypsin preventing autodigestion. In inherited pancreatitis (right) mutations in *PRSS1 *or *SPINK1 *lead to an imbalance of proteases and their inhibitors resulting in autodigestion. The role of CFTR is until now poorly understood. Abbreviations: AP = activation peptide (According to Witt, [85]).

Thus, all pancreatitis associated *PRSS1 *mutations discovered to date seem to cluster in the N-terminal half of the molecule encoded by exons 2 and 3. It is important to note, however, that investigation of the *PRSS1 *gene in patients with suspected genetically determined chronic pancreatitis is restricted to these exons in most laboratories and possible C-terminal mutations may have been missed. The discovery of pancreatitis associated cationic trypsinogen mutations in 1996 demonstrated that trypsinogen plays a central role in the pathogenesis of human pancreatitis. These mutations seem to disturb the balance of proteases and their inhibitors within the pancreas leading to autodigestion of the organ (Figure 3).

The R122H and the N29I mutations are the most common *PRSS1 *mutations worldwide. They have been frequently reported from Europe, North America and Asia [56] and R122H was also recently found in a family of Aboriginal descent in Australia [57]. Neither mutation was detected in two hereditary pancreatitis families from Brazil [58] and no hereditary pancreatitis cases have been reported from Africa. Here we summarize the most important genetic and biochemical data of the two common HCP associated *PRSS1 *mutations N29I and R122H. These data are more extensively discussed in an actual review by our group [59].

### R122H and increased trypsin stability

David Whitcomb proposed that the Arg122-Val123 autolytic peptide bond in trypsin plays an important role in the degradation of prematurely activated trypsin in the pancreas. Destruction of this "failsafe mechanism" by the R122H mutation would increase intrapancreatic trypsin activity and disturb the protease-antiprotease equilibrium and eventually provoke pancreatitis [5]. Biochemical evidence supports the notion that Arg122 is important for autolysis of trypsin and mutations of this amino-acid result in increased trypsin stability [60-63]. A study using cerulein-induced zymogen activation in isolated rat acini demonstrated that autodegradation of trypsin mitigates cathepsin B-mediated trypsinogen activation, suggesting that a failsafe mechanism might be indeed operational in the mammalian pancreas [64]. The Whitcomb model in its original form has remained very popular over the years, even though more detailed biochemical analysis indicated that the R122H mutation results not only in increased trypsin stability but also in increased zymogen stability and increased autoactivation [62,63]. A weak trypsin-inhibitory activity associated with the Arg122 site is also lost in the R122H mutant [63]. Thus, the pleiotropic biochemical effect of R122H raises the possibility that the pathogenic alteration is unrelated to trypsin stability. More importantly, the model fails to explain how the other pancreatitis associated *PRSS1 *mutations might work, as the majority of these do not affect trypsin stability.

### N29I and enhanced trypsinogen autoactivation

Biochemical characterization of the N29I mutation using recombinant trypsinogen found no effect on trypsin or trypsinogen stability. On the other hand, two independent laboratories observed moderately increased autoactivation in 4 studies [62,65-67]. The N29T mutant, firstly described by Pfützer et al. in 2002, exhibited a phenotype similar to that of R122H, both increased trypsin stability and enhanced autoactivation were documented [11,64]. Because increased autoactivation was observed with the R122H, N29I and N29T mutations, whereas N29I had no effect on trypsin stability, the logical conclusion was put forth that enhanced autoactivation is the common pathogenic mechanism of hereditary pancreatitis associated *PRSS1 *mutations [62].

### Animal models

Genetically engineered animals offer the opportunity to study the effects of the foresaid mutations in vivo.

Transgenic expression of rat SPINK1 in mice reduced the severity of experimental secretagogue-induced pancreatitis [68]. The transgenic animals had 190% increased endogenous trypsin inhibition capacity. Transgenic expression of SPINK1 did not hinder trypsinogen activation, but reduced trypsin activity after supramaximal stimulation with cerulein. These in vivo results underline the hypothesis that enhanced inhibitory capacity of trypsin protects against pancreatitis.

Results from a mouse with targeted disruption of the pancreatic secretory trypsin inhibitor are puzzling [69]. A knockout (-/-) of the mouse homologue of human SPINK1, murine Spink3, is lethal within two weeks after birth. Spink3 -/- embryos developed normally until day 15.5 after conception. Subsequently, autophagic degeneration of the pancreatic acinar cells started, interestingly without significant inflammatory cell infiltration. In this study, the authors were not able to detect enhanced trypsin activity in the acinar cells of the SPINK -/- animals. However, in a further study enhanced tryptic activity was found in pancreatic acini prepared one day after birth using a more sensitive assay [70]. Therefore, these data indicate that the total loss of SPINK3 function leads to a strong imbalance in favour of trypsin activity resulting in acinar cell death and involution of the whole gland and finally leading to the lethal phenotype.

Two recent publications describe transgenic animals expressing R122H mutated trypsinogen [71,72]. Our group developed a mouse expressing R122H human trypsinogen in the exocrine pancreas by using the rat elastase-2 promoter [71]. The animals showed slightly elevated serum levels of lipase without any significant histological alteration, suggesting a subtle acinar damage. After repetitive induction of experimental pancreatitis, pancreata of transgenic animals showed a higher inflammatory reaction than controls. The mild phenotype in these animals is probably caused by a low expression level of R122H mutated trypsinogen.

Transgenic expression of the R122H mutation of murine trypsin 4 in mouse pancreas led to progressive fibrosis and chronic inflammation of the pancreas [72]. Repetitive inductions of experimental pancreatitis with supramaximal doses of cerulein resulted in extensive deposition of collagen in periacinar and perilobular spaces of the transgenic animals. Thus, this animal model, which gives a significant expression level of R122H mutated trypsinogen, seems to recapitulate the human disease.

In summary, the animal studies indicate that disarranging the balance between trypsin and its inhibitors in favour of a higher intra-acinar tryptic activity contributes to the development of pancreatitis.

### The trypsinogen und *SPINK1 *mutation database

Since the first description of a trypsinogen mutation in hereditary pancreatitis, the experimental and clinical information on genetic alterations in chronic pancreatitis has been rapidly growing, resulting in a more and more complex data set. To address this issue, we implemented a continuously updated database in early 2001, which contains all genetic variants of the *PRSS *and *SPINK1 *genes [73]. In addition to exact genetic data, this database contains links to the clinical characterization of patients with different mutations and to *in vitro *studies with mutant molecules.

### Molecular diagnostic methods

The recommended "gold standard" method is direct DNA sequencing of both strands. Most laboratories have focused their studies on *PRSS1 *exons 2 and 3, and until now no unambiguous disease associated mutation has been identified in the other exons. However, it is still possible that new variants will be identified in exons 1, 4 and 5, or in the intronic and promoter regions. Interestingly, the triplication of a segment containing the *PRSS1 *gene was actually found in certain patients with HCP. This triplication seems to result in a gain of trypsin through a gene dosage effect and represents a previously unknown mechanism causing HCP [74].

There are several more methods to detect *PRSS1 *mutations such as single strand conformation polymorphism analysis (SSCP), restriction fragment length polymorphism analysis (RFLP) or denaturing high performance liquid chromatography (DHPLC). These methods are limited by a lack of sensitivity and specificity and since the nucleotide sequence is not detected subsequent sequencing of the altered probe is necessary in most cases. Above all Howes et al. showed that false negative results may be obtained regarding the R122H mutation, if RFLP is used with the restriction endonuclease *Afl III*, when a neutral polymorphism is present within the restriction site [75]. Melting curve analysis using fluorescence resonance energy transfer (FRET) probes is a highly efficient method with a very high sensitivity and specificity, however, only detecting the defined mutation and only partly other mutations located under the probes.

Therefore, since DNA sequencing has become more affordable within the last years and is also able to detect further mutations within the amplified fragment, sequencing of both strands should be performed.

### Differential diagnosis

Well-recognized causative factors of chronic pancreatitis are anatomic anomalies, metabolic disorders, trauma, cystic fibrosis and inflammatory bowel disease. Since HCP manifests predominantly in childhood or early adulthood, alcohol abuse as the most common predisposing condition can nearly be ruled out. One of the most important differential diagnoses is cystic fibrosis. Therefore, all patients with onset of the disease in childhood and early adulthood should be screened for a pathological sweat chloride test and subsequently for the most common *CFTR *mutations of their population. Other rare differential diagnoses are hyperlipidaemia type I, familiar (hypocalciuric) hypercalcaemia (FBH), hereditary hyperparathyroidism and autoimmune pancreatitis, last-mentioned usually manifesting in late adulthood [76-80].

### Genetic counselling and testing

First of all, genetic counseling should be performed in an experienced multi-disciplinary clinic that can address the resulting issues. Before genetic testing is performed implications of finding HCP related mutations in the *PRSS1 *gene for the health and the medical care of the patients should be discussed. Moreover, the elevated pancreatic cancer risk and the possible adverse effects for the patient regarding health and life insurance and employment should be brought up. Before performing the test, the form of communicating the test result should be assessed. Genetic testing should only be performed after informed consent.

The indication for *PRSS1 *and *SPINK1 *mutation testing in symptomatic patients should be one of the following:

1. recurrent unexplained attacks of acute pancreatitis and a positive family history

2. unexplained chronic pancreatitis and a positive family history

3. unexplained chronic pancreatitis without a positive family history after exclusion of

other causes (see differential diagnoses)

4. unexplained pancreatitis episode in children

Noteworthy, genetic testing in children is a complex issue, since depending on their age children cannot always be included in the process of decision making whether genetic testing should be performed. Therefore, extensive genetic counseling, illuminating the above-mentioned aspects is necessary. Another important facet may be the information for anxious parents that genetic testing cannot predict the age of onset or the severity of the disease and that the findings of the analysis do not change the management of the disease today.

Beyond the hitherto discussed aspects the detection of *PRSS1 *and *SPINK1 *mutations will lead to a correct classification of the disease, helping the affected individual to better understand their disease and clearing out misclassifications (e.g. alcoholic chronic pancreatitis).

Predictive genetic testing should only be offered by a recognized service with adequate pre-test counselling, post-test support and clinical follow up. The persons capable of testing should have a first degree relative with a defined HCP gene mutation, should be able to understand the different above mentioned aspects and the request for genetic testing should have been consistently stated [81].

We want to emphasize anew that all HCP patients or potential *PRSS1 *mutation carriers should be informed that the finding of a disease associated mutation does neither predict the onset or course of the disease nor affords specific diagnostic or therapeutic consequences.

### Antenatal diagnosis

As the penetrance of inherited *PRSS1 *mutations is incomplete, and a highly variable disease manifestation occurs within the most families, no antenatal diagnosis should be encouraged. Even in recently published guidelines concerning genetic testing in HCP the authors had reservations against antenatal diagnosis, but highlight that they cannot be so prescriptive as to refuse molecular genetic testing in an age of patient autonomy and informed consent [81]. Requesting parents should be informed, that even a painful course of the disease is self-limited to only a few years in the most cases. No patient in our cohort find his life not lifeable due to disease-associated health limitations.

### Management including treatment

No prospective randomised trial has been published for any medical or surgical problem in the management of HCP. Several case reports, but few systematic studies, address the medical and surgical treatment of HCP. Generally, the treatment does not differ from common forms of chronic pancreatitis.

In most instances treatment of chronic pancreatitis focuses on pain management, maldigestion, diabetes, pseudocysts, bile duct obstruction, duodenal obstruction and pancreatic cancer [27,82-84]. Regarding pain management amenable causes like pseudocysts, biliary and duodenal obstruction, and coincident peptic ulceration should be ruled out before an adequate pain therapy is started.

To stop the progression of the disease the consumption of alcohol should be an infrequent event, whereas smoking should be avoided completely. In concern of pancreatic insufficiency, frequent small meals with a low fat content may help to limit pancreatic stimulation while maintaining caloric intake. Maldigestion should be treated by supplementation of pancreatic enzymes in sufficient dosage. Management of endocrine failure is carried out according to the guidelines of approved diabetes societies.

A widely accepted indication for surgery in patients with HCP is chronic pain in the presence of a persistent dilatation of the main pancreatic duct. Many surgeons favour a longitudinal pancreaticojejunostomy (Puestow procedure modified by Partington and Rochelle) with good early results.

However, in patients without pancreatic duct dilatation, the operation seems not to be beneficial for the further course of pancreatitis or for the quality of life [85]. In addition, surgical interventions should be discussed with caution in children, as periods of only a few or even no symptoms frequently extend into adulthood. For these reasons, a decision to wait and see might be more appropriate than early operation in paediatric patients with HCP. In adults, surgical decisions on the basis of the guidelines concerning the indications for surgical treatment of patients with chronic pancreatitis of the more common underlying causes seem to be as effective in patients with HCP as in patients with chronic pancreatitis of other origins [86]. Unfortunately, there are no data regarding endoscopic procedures in the management of chronic pancreatitis patients. Therefore, prospective studies are needed to further elucidate the role of surgical and endoscopic management in chronic pancreatitis.

### Prognosis

Today, the individual prognosis of HCP is unpredictable. It is neither possible to predict further episodes of acute pancreatitis, chronic bile duct obstruction, exocrine or endocrine pancreatic insufficiency nor the individual risk of pancreatic cancer.

### Unresolved questions

From a genetic aspect, a more interesting quest in the near future will be for modifier genes that might explain the incomplete penetrance i.e. why some carriers of *PRSS1 *mutations remain healthy, whereas their relatives with the same mutation exhibit severe disease. Despite intensive research, the disease mechanism remains poorly understood. The biochemical alterations caused by the mutations have been mostly clarified *in vitro*, but their phenotypic effect *in vivo *in most instances remains unclear. In this respect, future development of cellular and animal models of HCP will be particularly valuable.

## Competing interests

The author(s) declare that they have no competing interests.

## Note

Some parts of this work have been published in "Human Mutation" [59].
